# Potential Immunomodulatory Effects from Consumption of Nutrients in Whole Foods and Supplements on the Frequency and Course of Infection: Preliminary Results

**DOI:** 10.3390/nu13041157

**Published:** 2021-04-01

**Authors:** Ewelina Polak, Agnieszka Ewa Stępień, Olga Gol, Jacek Tabarkiewicz

**Affiliations:** 1 Department of Dietetics, Institute of Health Sciences, College for Medical Sciences, University of Rzeszow, al/mjr. W. Kopisto 2a, 35-310 Rzeszów, Poland; astepien@ur.edu.pl; 2 Centre for Innovative Research in Medical and Natural Sciences, College for Medical Sciences, University of Rzeszow, Warzywna 1A, 35-310 Rzeszów, Poland; jtabarkiewicz@ur.edu.pl; 3 Department of Human Immunology, Institute of Medicine, College for Medical Sciences, University of Rzeszow, Warzywna 1A., 35-310 Rzeszow, Poland; olga.gol92@gmail.com

**Keywords:** immunomodulatory factors, immune system, nutrients, probiotics, omega-3 fatty acid

## Abstract

A diet rich in nutrients should be implemented in order to boost the immune system and prevent infections. To investigate which nutrients are commonly consumed, an anonymous survey was given to 120 individuals and their responses were collected. The respondents answered questions relating to their health status, and their consumption of nutrients and supplements that produce immunomodulating effects. The participants were also asked about any prior viral, bacterial or fungal infections experienced, and in particular, infection frequency, course, and duration. The data collected were subjected to a statistical analyses to assess the relationship between the reported frequency of infections and nutrients consumed including vitamins D3, A, C, E, selenium, zinc, iron, β-carotene, omega-3 fatty acids as well as live active probiotic bacteria. The findings show that vitamin and mineral supplementation did not positively affect the duration, frequency, or course of infections in the surveyed sample. An exception was vitamin D3 supplementation that was correlated to sporadic incidence of viral infections. Conversely, immunity was positively affected by consumption of natural nutrients contained in whole food (vitamin C, iron, selenium, omega-3 fatty acids), evidenced by lower incidences and milder courses of infection.

## 1. Introduction

Specialists in medical and nutritional sciences study the possibility of modulating and controlling the immune responses of the body. The term immunomodulation refers to interventions which induce specific changes in the immune system, irrespective of the body’s health or nutritional status. The substances contained in food that affect the immune system are called immunomodulators and may both stimulate and suppress specific and non-specific mechanisms of the immune response [1].

Immunomodulators include vitamins A, C, D3, E, and β-carotene as well as microelements such as zinc, selenium, iron, omega-3 fatty acids, and live active probiotic bacteria. For example, adequate dietary uptake of β-carotene contributes to a stronger cellular response by increasing the number of monocytes which produce MHC II molecules [2,3]. Insufficient dietary uptake of β-carotene (as provitamin A) from orange, yellow, and green-colored vegetables, adversely affects functioning of the immune system and contributes to an increased incidence of infectious diseases [4]. This may also be associated with the role of vitamin A in maturation and proliferation of lymphocytes, monocytes and neutrophils. In the case of vitamin A deficiency, the neutrophil count is normal, but the cells have weaker activity. Individuals with low level of retinoids were found to have a lower number of CD4^+^ cells, and a lower CD4^+^ to CD8^+^ cell ratio. Furthermore, retinoid deficiency adversely affects the phagocytic activity of monocytes [1,2]. Vitamin C, as an immunostimulant, affects intracellular nucleotide pools, prostaglandin synthesis and production of cytokines. It also inhibits activity of histamine and stabilizes activity of the enzyme 5-lipoxygenase. Moreover, vitamin C stimulates production of interferon and increases immunity in vivo by affecting the activity of NK cells as well as T and B lymphocytes. Ascorbic acid is also used for treatment of some disorders linked to abnormal functioning of phagocytes [5,6]. Vitamin D also affects the immune system. The active metabolite of vitamin D, calcitriol (1,25-dihydroxycholecalciferol), leads to a shift in the production of cytokines from Th1 to Th2, and induces a decreased expression of co-stimulatory molecules. Vitamin D3 stimulates proliferation of monocytes and monocytic lineage cells in mature form and deficiency of this vitamin impairs the process, consequently damaging chemotactic potential and the phagocytotic capacity of macrophages. Furthermore, calcitriol regulates the activity of antigen-presenting cells (APC) [2,7,8]. Research has shown that calcitriol synthesis in dendritic cells functions to suppress excessive reactivity of the immune system. As to the effects of vitamin D on B cells, it has been shown that vitamin D3 (1,25(OH)2D3) inhibits differentiation of new B cells and production of antibodies but does not affect existing ones. Increased expression of vitamin D receptors (VDR) is also observed on T lymphocytes as their activation increases. After the active metabolite of vitamin D3 connects with VDR, there is a change in the nature of the cytokines secreted on T cells, whereby activation of effector T cells is suppressed and regulatory T cells are stimulated [2,9]. Twenty percent of vitamin D is supplied by food. The products with the highest amounts of vitamin D are sea fish (mackerel, salmon, herring) and fish oils (5–10 µg and over 10 µg) [1].

Research has shown that vitamin E administered to rodents led to a decreased concentration of IL-1β, and increased levels of IL-2 and IL-4. Vitamin E was also reported to modulate the balance between Th1 and Th2 lymphocytes [5]. The richest sources of tocopherol are wheat germ oils (133 mg per 100 g) and sunflower seeds (49 mg per 100 g). γ-tocopherol (60 mg per 100 g) appears in soybean and corn oil in greater amounts than α-tocopherol [1]. Normal functioning of immune cells depends largely on availability of iron from foods. Anemia resulting from deficiency of this element increases the risk of infection due to weaker granulocyte activity (chemotactic performance and activity of myeloperoxidase), and a lower activity of macrophages and NK cells contributing to a decrease in the number of T cells and impairment of differentiation processes. The findings of studies involving mouse models and human subjects suggest that under conditions of iron deficiency, monocytes and macrophages produce lower amounts of proinflammatory cytokines IL-6 and TNF-α in response to infection [10,11]. Selenium is another microelement which affects functioning of the immune system. It is contained in the amino acids selenomethionine and selenocysteine which are used by the body to build strong antioxidant enzymes (e.g., glutathione peroxidases and thioredoxin reductases). Selenium affects the humoral and cellular immune response by interacting with non-specific macrophages as well as T and B lymphocytes slowing the process of T cell proliferation and impacting the activity of NK cells. Insufficient selenium uptake is associated with predominant production of Th2 lymphocytes and proinflammatory M1 macrophages while normal selenium supply leads to production of Th1 lymphocytes and proinflammatory M2 macrophages [12]. 

Zinc is important for cell sustenance and growth in both the innate and adaptive immune systems. It is involved in protection against oxidative stress. Zinc deficiency slows development, activation and maturation of lymphocytes. It is also a necessary cofactor for thymulin which modulates secretion of cytokines. Individuals with zinc deficiency, particularly children, are susceptible to respiratory and digestive system infections [13,14]. The above-mentioned elements are found in animal products such as meat and seafood. They can also be found in vegetables, nuts and grains [1,15].

Immunomodulatory properties have also been attributed to omega-3 fatty acids (α-Linolenic acid, EPA and DHA) and omega-6 fatty acids (linoleic acid and γ-linolenic acid). Inflammation inhibitors are produced in the body during synthesis of polyunsaturated fatty acids (PUFAs). The immunostimulatory activity of fatty acids is associated with modified production of eicosanoids (leukotrienes, prostaglandins, thromboxane) and largely depends on the type and quantity of fatty acids in the diet [1].

Probiotic bacteria are a significant part of the large gut flora with an estimated 1014 cells. They are a component of gut associated lymphoid tissue (GALT). Gut associated lymphoid tissue is particularly important for the functioning of the immune system because it comprises 75% of the lymphoid cells of the entire immune system, and approximately 50% of the lymphocytes; furthermore, it produces approximately 80% of all immunoglobulins (particularly IgA) [1]. Probiotic bacteria affect the humoral and cellular immune response occurring in the gastrointestinal tract mucosa. The anti-inflammatory effects of their activity are linked with the fact that they promote normal functioning and differentiation of T and B lymphocytes, dendritic cells and macrophages. The latter, owing to involvement of probiotic bacteria, increase the activity of antigen presentation to B lymphocytes and production of IgA [16]. Moreover, numerous studies have shown that probiotic bacteria can modulate the process of cytokine production. Many strains of Lactobacillus stimulate secretion of IFN-γ and IL-12 by Th1 lymphocytes which are associated with cytotoxic activity of NK cells. Bifidobacterium and Lactobacillus affect expression of the anti-inflammatory and regulatory cytokines TGF-β and IL-10 which suppress Treg lymphocytes [17]. Probiotics are present in supplements and fermented products such as sour dairy or fermented vegetables. Fermented foods have been shown to provide many health benefits having antioxidant, anti-bacterial, anti-fungal, anti-inflammatory, anti-diabetic and anti-atherosclerotic effects related to the immune system [18].

By introducing food to a diet that is rich in immunomodulators, it is possible to impact functioning of the immune system. For example, a change from refined grain products to whole-grain foods decreases levels of free radicals circulating in the body as well as the levels of pro-inflammatory cytokines such as IL-6, IL-18 and TNF-α. Notably, decreased uptake of fat, particularly saturated fatty acids, reduces c-reactive protein (CRP) levels in patients with metabolic syndrome. In view of the above information, the Mediterranean diet model offers the optimum strategy for treatment of diseases whose pathogenesis includes inflammation. Most importantly, this dietary model contains numerous sources of vitamins, minerals, flavonoids and unsaturated fatty acids [19]. 

## 2. Materials and Methods

This survey-based study involved 120 individuals (78 women and 42 men). The participants ranged in age from 17 to 35 years, with the largest group comprising individuals aged between 21 and 25 years (86%). The mean age was 23.0 ± 2.40 years. The respondents voluntarily agreed to participate in the study.

### 2.1. Study Design

**Figure 1:**
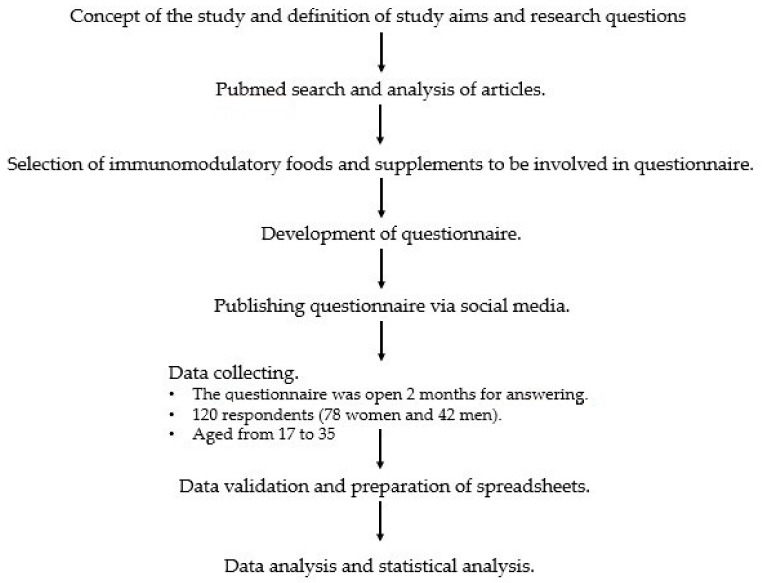
Chart of study design.

The research method applied was based on diagnostic polling carried out with use of a survey. The survey was created using Google forms and published on social media. The first part of the questionnaire consisted of 25 closed-ended questions requiring one response to be selected. The second part was in a tabular form concerning the frequency with which the respondents consumed specific groups of foods and dietary supplements containing nutrients that affect immunity. The specific groups of foods and dietary supplements were selected on the basis of reviews of scientific articles in Pubmed (pubmed.ncbi.nlm.nih.gov (accessed on 25 January 2021)) found by combinations of two search terms: food and immunomodulatory or immunomodulatory and supplements. The survey also included questions related to the current health status of the respondent. The survey participants were asked to specify how often they were affected by viral, bacterial and fungal infections, and to report the duration and course of such infections. The study design is surmised in Figure 1.

### 2.2. Statistical Analysis

Responses were acquired using an electronic survey questionnaire. Responses were collected and analyzed using Excel 2010 (Microsoft, Redmond, WA, USA) and Statistica 12.5 software (StatSoft, Kraków, Poland). These were applied to calculate percent rates and cumulative percentage values, and to perform statistical analyses assessing significance of categorical variables with Pearson’s chi-squared test. Multi-way tables were applied to sets of categorical data to evaluate the likelihood that any observed difference between the sets arose by chance. Yates’s correction for continuity was used when the number of observations in any field in contingency table was lower than 5. Significance was assumed at *p* < 0.05. 

## 3. Results

The results showing self-reported incidences of viral, bacterial and fungal infections, duration and course of infections are reflected by the data presented in Table 1.

Out of all the respondents, 36.7% declared they were using dietary supplements particularly designed to improve immunity. The frequency of respondent food or supplement uptake with a high content of immunomodulators is shown in Table 2.

The percentage of respondents that declared common daily consumption of specific nutrient rich foods was iron (39.2%), vitamin C (39.2%), beta-carotene (31.7%), vitamin A (28.3%) and probiotic bacteria (25%). At the same time, as many as 53.3% of respondents stated that they do not eat iron-rich foods at all. The least consumed supplements are those containing selenium (67.5%), zinc (60.0%), beta-carotene (60%) and vitamin E (53.3%). The most frequently taken supplements were vitamins C and D. Vitamin C was taken daily by 12.5% of people, and vitamin D by 11.7%. 

Respondents consuming vitamin C rich products on a daily basis reported a low incidence (1–3 times per year) of viral infections (*p* < 0.001) compared to individuals with less frequent consumption. A similar effect was found in respondents consuming foods with a high content of iron several times per week (*p* < 0.001).

The findings show that dietary factors also affect the course of infection. Mild infections were reported by individuals regularly consuming foods with high contents of omega-3 fatty acids (*p* < 0.04), vitamin C (*p* < 0.001) and selenium (*p* < 0.05) more than a few times per week.

Supplementation of vitamin D3 (daily or more than a few times per month) was associated with self-reported occasional incidence (1–3 times per year) of viral infections (*p* < 0.001).

Entirely different correlations were found when the remaining supplements were taken into account. Notably, respondents who reported a low incidence of viral infections (1–3 times per year) used supplements intended to improve immunity only rarely (*p* < 0.05). This may be the reason for the relationship between low incidence of viral infections reported by the participants who also rarely (less than a few times per year) consumed supplements containing omega-3 fatty acids (*p* < 0.05), strains of probiotic bacteria (*p* < 0.05), iron (*p* < 0.05) or selenium (*p* < 0.001). 

A significant relationship was identified between infrequent use of iron supplementation (never and/or less than a few times per year) and short duration of infections lasting 4–6 or 1–3 days (*p* < 0.05). 

A mild course of infection was also reported by respondents who never or only occasionally (less than a few times per year) took supplements containing zinc (*p* < 0.01), beta-carotene (*p* < 0.05) and vitamin A (*p* < 0.01).

We also analyzed the differences between respondents according to their gender. We didn’t find any statistical differences in the subjective evaluation of health status. On the other hand, women declared general infections more frequently (*p* < 0.05). This could be associated with a higher frequency of urinary tract infections in the female population. Women also declared use of immunostimulative supplements more frequently than men (42.31% vs. 26.19%, respectively) but this difference was not significant (*p* = 0.07). Male respondents declared eating iron rich food e.g., red meat more frequently (*p* < 0.05). Among male participants, 57.14% declared eating meat every day in contrast to 29.49% of women. Women also declared eating beta carotene and vitamin E rich foods more frequently, but this difference was not statistically significant (*p* = 0.07 and *p* = 0.05, respectively).

## 4. Discussion

According to available data, rates of the use of dietary supplementation range from 54% up to 82% [20]. Supplements designed to boost immunity constitute a significant part of all supplements taken. In the present study, 36.7% of the respondents reported they took this type of supplementation. In another study of young people aged 19–26 years, approximately 23% of the survey participants reported they were taking supplements affecting the immune system [21]. On the other hand, a study investigating varied age groups reported that immunity-boosting supplements were taken by approximately 41% of respondents [22]. There are a small number of research studies that have focused on a large number of immunomodulators in both supplementation and in foods. Our study is the first to take into account such a large number of substances and their impact on viral, bacterial and fungal infections. Strengthening the immune system has always been important, and in view of recent events, it is receiving greater attention. 

According to a study by Skop-Lewandowska et al., interviewed participants aged between 20 and 35 years reported consuming milk and milk-based drinks, vegetables and fruits daily. These products are good source of vitamin C, beta-carotene (pro-vitamin A) and probiotic bacteria. Sources of iron such as fish and meat were eaten once a week [23]. Supplements taken most commonly are those containing vitamins C, E, D, and minerals such as calcium and iron. It should be mentioned that studies of the frequency of consumption of dietary supplements do not usually focus on those that contain immunomodulators [24,25].

According to a study by Zegan et al., the nutrient most commonly used as a supplement to boost immunity was vitamin C. In this study, it was taken by 19% of the survey participants [22]. Current findings also show that vitamin C is also the most frequently supplemented vitamin; it was taken on a daily basis by 12.5% of the respondents. Numerous studies investigating the impact of vitamin C on the human body, in line with their design, have implemented vitamin C supplementation. One must remember, however, that high contents of this vitamin are found in certain foods and therefore uptake may easily be augmented by using natural sources and this fact should be taken into account while interpreting research findings. Analysis of the frequency with which vitamin C rich supplements and foods are consumed in relation to infections experienced shows that it is vitamin C contained in natural foods rather than vitamin C supplements that affect the incidence of bacterial, viral and fungal infections and the course of bacterial infections. This may be linked to the latest research reports that suggest that healthy individuals possibly do not benefit from supplementation if their daily dietary vitamin C uptake amounts to 200 mg or more [26]. However, when the diet lacks this vitamin, the deficiency is manifested by a greater risk of upper respiratory tract infections and pneumonia [27]. During an existing infection, the level of oxidative stress is elevated. The mechanism of its development is not known in detail but the antioxidant activity of some nutrients including vitamin C, beneficially affects infection duration. Notably, adequate level of vitamin C in the body decreases inflammation and positively affects endothelium integrity, leading to increased immunity to infection [26]. In a study involving 3258 male subjects, it was shown that high content of vitamin C in the diet comprising its natural sources was correlated with lower CRP levels in the blood, which reflects its anti-inflammatory properties [28]. 

Supplementation of vitamin D3 is recommended by numerous scientific societies and its impact on the immune system is well known. This is largely linked with the fact that receptors of this vitamin occur on immune system cells [29]. A meta-analysis showed that supplementation of vitamin D prevents respiratory tract infections, particularly those of viral origin [30,31]. Likewise, in the present study, frequent supplementation of vitamin D3 decreased the incidence of viral infections. Other studies show that low levels of vitamin D3 in blood contributes to respiratory infections, and the greatest benefits from supplementation are observed in individuals with deficiency of this vitamin [32,33]. It was also shown that the risk of acute respiratory tract infections decreased by 12% in individuals taking vitamin D3 supplements, irrespective of the dose, compared to individuals who did not use such supplements [31]. It is speculated that respiratory tract infections are most common in winter because of the low levels of vitamin D3 in blood resulting from lower exposure of skin to sunlight promoting production of the vitamin by the body [34]. 

The functioning of the immune system is also affected by polyunsaturated omega-3 fatty acids because of their anti-inflammatory activity. Importantly, inflammation is a key element of the immune response and it is induced in order to eliminate infections. Various pro-inflammatory mediators are produced by several different types of cells of the immune system. The process of inflammation subsides at the end of the immune response due to the activation of specific negative feedback mechanisms. It is this process that requires the omega-3 fatty acids eicosapentaenoic acid (EPA) and docosahexaenoic acid (DHA). When present at the site of inflammation, they are enzymatically transformed into specialized pro-resolving mediators (SPM). These molecules coordinate inhibition of inflammation and promote recovery during respiratory tract infection [35]. In the present study, frequent intake of omega-3 fatty acids was associated with a mild course of an existing viral infection. It should be pointed out that deficiency of omega-3 fatty acids in the diet may result in delayed resolving of inflammation or a severe course of the condition [36]. Research based on animal models showed that SPMs formed from EPA and DHA protect against acute lung injury and respiratory failure [14]. Research shows that omega-3 fatty acids in the form of fish oil, produce strong anti-inflammatory effects when added to the diet [37,38]. However, according to some studies, excessive levels of fish oil in the diet adversely affects the lymphocytic response, which may result from over supplementation. The addition of linseed oil in an amount corresponding to 15 g ALA per day resulted in a significant reduction in the proliferation of lymphocytes in the blood [2]. Hence, the effectiveness of omega-3 fatty acids largely depends on the dose and individual characteristics of an organism [37].

Iron deficiency impairs the immune response of the body while an excess also produces adverse effects. Sufficient amounts of this element in the diet is of key importance for a few immunity-related functions of the body, including differentiation and proliferation of T cells and production of reactive oxygen species (ROS), which destroy pathogens. However, excessive iron uptake, linked with supplementation, may lead to a greater risk of infection [39]. This occurs because most infection-causing factors also need iron for replication and survival [40,41,42]. Notably, in the current study, respondents who rarely experiencing viral infections reported they frequently consumed foods with a high content of iron but only rarely or never used iron supplements. 

Selenium deficiency leads to increased susceptibility to infection and is associated with higher levels of proinflammatory cytokines in various tissues. Selenoproteins are important for antioxidant mechanisms in the body; they affect the functions of leukocytes and NK cells [43]. Consumption of foods with high contents of selenium is necessary for normal functioning of the immune system. Selenium supplementation mainly produces an immunostimulating effect, particularly if supplementation compensates for a deficiency of this microelement. In such situations, it enhances cellular immunity and improves the immune response to viruses. On the other hand, excessive intake of selenium may lead to adverse effects as shown in research on animal models. In a study of mice, selenium supplementation exacerbated existing allergic asthma and weakened immune response to parasites [44,45,46]. 

The relationship between the occasional intake of supplements with vitamin A and a mild course of infection may result from the small number of individuals using this type of supplements among respondents who get ill sporadically. It is believed that vitamin A is an anti-inflammatory factor, contributing to improvement of the immune system and integrity of mucosa which protects the body against infections [47]. Research has shown that an adequate intake of vitamin A may lead to decreased mortality and morbidity in infectious diseases such as measles, pneumonia, human immunodeficiency virus (HIV) infection, and malaria [48]. Children with measles who took large doses of vitamin A supplements presented a higher IgG response to measles virus and a larger number of circulating lymphocytes [49]. The above information also indirectly relates to beta-carotene, which as a provitamin, is processed into vitamin A in the body. 

Zinc supplementation improves the mechanisms of innate immunity (e.g., phagocytosis by macrophages and neutrophils, NK cells activity), response of antibodies, and number of cytotoxic cells CD8þT [13]. Zinc is necessary for development, differentiation and activation of T lymphocytes [50]. Zinc deficiency adversely affects the immune system by reducing the number of macrophages and monocytes and by increasing the level of oxidative stress. Despite extensive research, we still do not fully understand the molecular mechanisms through which zinc regulates the function of macrophages [13]. A few randomized studies showed that supplementation with low or moderate doses of zinc (ranging from 10 to 45 mg per day) in healthy elderly subjects improves a few functions of the immune system, such as increases in the number of cytotoxic T lymphocytes and NK cells, and ultimately reduces the incidence of infections [51,52,53]. The findings of this study related to sporadic zinc supplementation and a mild course of infection may also result from only occasional use of supplements intended to improve immunity in individuals rarely experiencing infections. It should be pointed out, however, that large doses of zinc may lead to copper deficiency, which paradoxically may contribute to an impaired immune response of the body to viral infections [54,55].

Probiotic supplements are effective if used regularly. There is no evidence showing that occasional use of such supplements produces long-lasting effects in the gut microbiota modulating immune response [56,57]. A study showed that probiotic supplementation continued for at least four weeks may effectively prevent infections of upper respiratory tract and digestive system [58]. Likewise, a study carried out in winter involving a group of 477 male subjects who had not been vaccinated against influenza, showed that the incidence of viral respiratory tract infections was 13.6% lower in the group using probiotic supplements compared to the control group [58]. Probiotic therapy is frequently seen as a preventive measure which accompanies intake of antibiotics that adversely affect the microflora of the digestive system; this may explain the statistically lower number of individuals who take probiotic supplements and only occasionally experience infections. It should also be mentioned that the consumption of antioxidant vitamins and minerals can influence the gut microbiome and indirectly influence the immune system through GALT. Steinert et al. suggested that consumed vitamins can modulate the microbiome, alter redox potential, and improve gut motor function and enhance immunity [59].

Of course, our study is not free from limitations. The first and most important limitation is the relatively small sample size. It is necessary to increase the number of respondents and include a wider age-range of participants. Another important limitation is the lack of questions concerning the frequency of antibiotic use which can affect the condition of the intestinal microflora and immunity. It should be noted that probiotics are most often recommended after antibiotic therapy. The composition of microflora, which is largely responsible for immunity, may also be influenced by other factors such as dietary fiber and vitamin or mineral supplementation and their content in food. This requires further research to strengthen our results. Nevertheless, our study presents sound data and interesting findings which may indicate directions of future research.

## 5. Conclusions

In summary, the most positive effect reflected in the course of infections, and the decreased incidence of viral, bacterial and fungal infections result from consumption of foods with a high content of vitamin C. Additionally, the incidence of viral infections is effectively decreased by frequent consumption of foods with a high content of iron and by supplementation with vitamin D3. The above information is important, particularly in light of the pandemic caused by SARS CoV-19. The findings also show that occasional use or no intake of supplements containing iron, vitamin A, beta-carotene, omega-3 fatty acids, selenium, zinc, and probiotics are related to less frequent viral infections or a milder course of an existing infection. This insight allows one to emphasize the importance of a properly selected diet to meets the needs of the body’s immune system. Immunity is improved in the long-term by eating a proper diet that includes rich sources of natural vitamin C like fruits and vegetables. Our findings emphasize the advantage of such a diet over supplementation. It is worth noting that the only supplementation required to protect the body against infection is recommended supplementation with vitamin D3.

## Figures and Tables

**Table 1:** Reported incidence, average duration and course of infections (%).

Incidence of Infection	Viral Infections	Bacterial Infections	Fungal Infections
Never	10.0	52.5	79.2
Occasionally (1–3 times a year)	80.0	40.8	18.3
Often (4–6 times a year)	9.2	6.7	2.5
Very often (more than 7 times a year)	0.8	0.0	0.0
**The course of infection**	**Number of respondents reporting the course of infection**		
Severe, but without complications	38.4		
Mild	60.8		
Severe, and with complications	0.8		
**Duration of infection**	**Number of respondents reporting the duration of infection**		
>7 days	17.5		
4–6 days	36.7		
1–3 days	45.8		

**Table 2:** Percentage of people consuming a supplement or a product with a specific immunomodulatory component (%).

Product or Supplement/ Frequency of Consumption	Daily	A Few Times a Week	Once a Week or Several Times a Month	Several Times a Year	Never
foods rich in omega-3	7.5	25.8	45.8	18.4	2.5
supplements rich in omega-3	7.5	9.2	10.0	22.5	50.8
foods rich in vitamin A	28.3	45.0	25.0	0.8	0.8
supplements rich in vitamin A	8.3	10.0	11.7	19.2	40.8
products rich in β-carotene	31.7	40.8	26.7	0.0	0.8
supplements rich in β-carotene	3.3	4.2	17.5	15.0	60.0
products rich in vitamin E	16.7	26.7	45.0	9.2	2.5
supplements rich in vitamin E	6.7	3.3	21.6	16.7	51.7
products rich in vitamin C	39.2	40.0	16.7	2.5	1.7
supplements rich in vitamin C	12.5	10.8	27.5	19.2	30.0
products rich in vitamin D	2.5	13.3	56.7	20.8	6.7
supplements rich in vitamin D	11.7	11.7	15.8	15.8	45.0
products rich in iron	39.2	35.0	22.5	2.5	53.3
supplements rich in iron	4.2	7.5	21.7	13.3	0.8
products rich in zinc	15.8	19.2	48.4	15.0	1.6
supplements rich in zinc	3.3	5.8	14.2	16.7	60.0
products rich in selenium	19.2	25.8	48.4	5.8	0.8
supplements rich in selenium	2.5	1.7	16.7	11.7	67.5
products rich in probiotic bacteria	25.0	38.3	30.8	5.0	0.8
supplements rich in probiotic bacteria	5.0	5.8	15.0	26.7	47.5

## Data Availability

The data presented in this study are openly available in OSF at doi:10.17605/OSF.IO/K7SZ9.
